# In-situ monitoring by Raman spectroscopy of the thermal doping of graphene and MoS_2_ in O_2_-controlled atmosphere

**DOI:** 10.3762/bjnano.8.44

**Published:** 2017-02-10

**Authors:** Aurora Piazza, Filippo Giannazzo, Gianpiero Buscarino, Gabriele Fisichella, Antonino La Magna, Fabrizio Roccaforte, Marco Cannas, Franco Mario Gelardi, Simonpietro Agnello

**Affiliations:** 1 CNR-IMM, Strada VIII, 5, Zona Industriale, 95123 Catania, Italy; 2 Department of Physics and Chemistry, University of Palermo, Via Archirafi 36 - Palermo, 90123, Italy; 3 Department of Physics and Astronomy, University of Catania, Via Santa Sofia 64 - Catania, 95123, Italy; 4 AteN Center, Universita’di Palermo, Viale delle Scienze, Ed.18 - Palermo, 90128, Italy

**Keywords:** two-dimensional (2D) materials, graphene, MoS_2_, Raman spectroscopy, thermal doping

## Abstract

The effects of temperature and atmosphere (air and O_2_) on the doping of monolayers of graphene (Gr) on SiO_2_ and Si substrates, and on the doping of MoS_2_ multilayer flakes transferred on the same substrates have been investigated. The investigations were carried out by in situ micro-Raman spectroscopy during thermal treatments up to 430 °C, and by atomic force microscopy (AFM). The spectral positions of the G and 2D Raman bands of Gr undergo only minor changes during treatment, while their amplitude and full width at half maximum (FWHM) vary as a function of the temperature and the used atmosphere. The thermal treatments in oxygen atmosphere show, in addition to a thermal effect, an effect attributable to a p-type doping through oxygen. The thermal broadening of the line shape, found during thermal treatments by in situ Raman measurements, can be related to thermal phonon effects. The absence of a band shift results from the balance between a red shift due to thermal effects and a blue shift induced by doping. This shows the potential of in situ measurements to follow the doping kinetics. The treatment of MoS_2_ in O_2_ has evidenced a progressive erosion of the flakes without relevant spectral changes in their central zone during in situ measurements. The formation of MoO_3_ on the edges of the flakes is observed indicative of the oxygen-activated transformation.

## Introduction

A wide interest for two-dimensional (2D) materials has grown in recent years [1]. Graphene (Gr) is a 2D carbon material with zero-energy band gap and turned out to be relevant because of its electrical, transport and optical properties. It is considered the lead example of the emerging 2D solids [2–4]. For example, optical transparency and bipolar charge carrier availability of Gr paved the way for studies and applications in many fields of materials science and technology including optics, electronics, and photovoltaics [5–6]. In these contexts, different growth processes optimized to obtain large area-samples, comprising epitaxial growth on silicon carbide [7–8] and chemical vapor deposition (CVD) on catalytic metals [9–10] followed by the poly(methyl methacrylate) (PMMA) assisted transfer [11–12], enlarged the interest and perspectives for applications.

In particular in view of the realization of electronic devices and to obtain Gr-based field effect transistors, one demanding physical feature of Gr connected to the energy levels distribution is the removal of the zero energy gap between valence and conduction states to enable a more efficient current modulation in the devices on/off states [13]. Furthermore, the need to control the charge carrier density and the energy of the Fermi level evidenced that doping of graphene is an important aspect for electronic devices. The possibility to obtain stable p- or n-type doping in order to modulate the Gr sheet resistance for specific electronic and optoelectronic applications is mandatory for technical application such as thin flexible circuits and interconnecting transparent conductive electrodes for solar cells and/or touch screens [6]. Among the possible dopants for Gr, an easily accessible and promising one is oxygen. It acts as a p-type dopant and can also be activated by thermal treatments [13–14]. Nevertheless, thermal activation has the drawback that it could induce relevant changes to the Gr structure because of the high annealing temperatures or reactions with ambient gas molecules [13–19]. We have recently shown [19] that p-type doping of Gr can be induced by thermal treatment in oxygen at temperatures below 400 °C. It has also been shown that the effect of doping is sensitive to the ambient atmosphere. In particular, water molecules affect the doping stability [20–21].

Alongside Gr, the transition metal dichalcogenide MoS_2_ is one of the stable 2D materials of interest [1]. Especially the possibility to produce van der Waals heterostructures combining Gr and MoS_2_, is pushing the study of this subject [1]. Indeed, the non-zero band gap, good chemical sensitivity and photo response of MoS_2_ pave the way for its application in optoelectronics, sensing and photovoltaic devices [22–24]. In this context, the possibility to tune the properties of MoS_2_ and to evaluate the thermal effects in the case of heterostructures are of interest [25–26]. As recently shown, the possibility to obtain doping by treatment in controlled atmosphere, accurately studied through Raman spectroscopy, could be useful for a future application of this material [25,27–28]. In fact, the A_1g_ Raman mode band at about 403 cm^−1^ undergoes a red shift and line width widening after n-type doping, and a blue shift and a line width shrinking after p-type doping, allowing for a spectroscopic control of the doping level [25,27].

In this paper, in order to clarify the dynamics of doping and the role of atmosphere during thermal treatment as well as to check the material stability, we investigated the effects of temperature and atmosphere by in situ Raman measurements during thermal treatment in air or in O_2_ gas on samples of Gr produced by CVD on copper foil and successively transferred on a SiO_2_ substrate on Si. Analogous studies were carried out on MoS_2_ transferred on the same substrate to evaluate the features and doping effectiveness of thermal processes similar to those used for Gr.

## Results and Discussion

**Figure 1:**
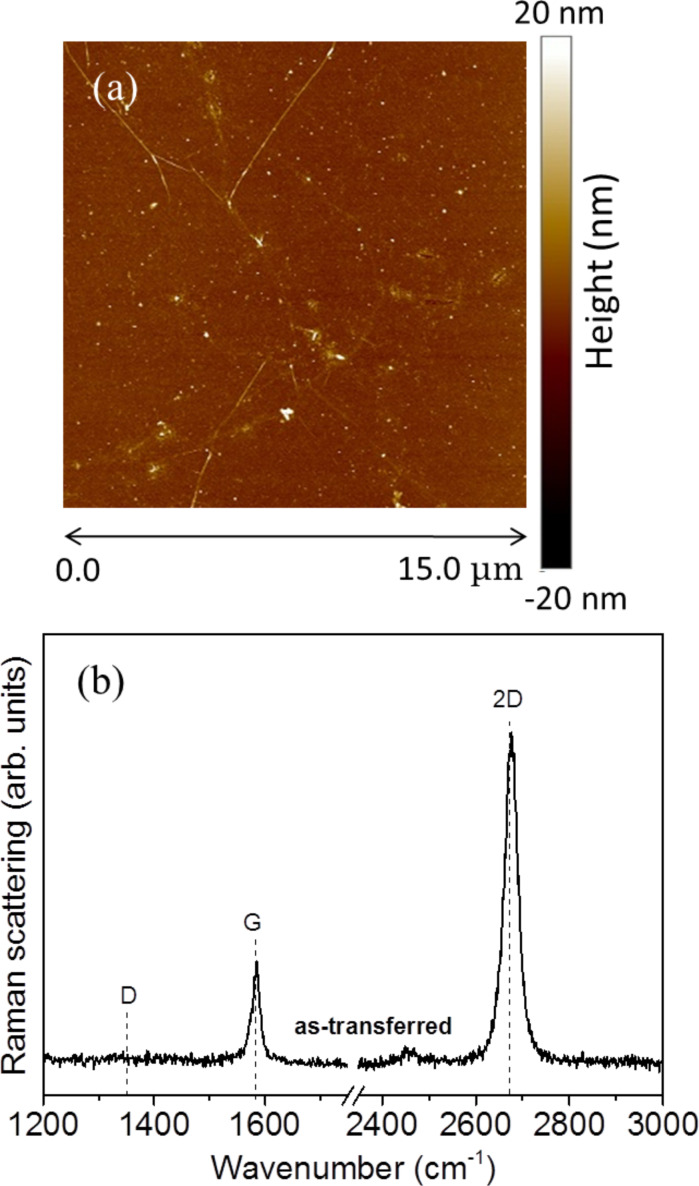
(a) AFM morphology image and (b) micro-Raman spectra of the as transferred graphene on SiO_2_ substrate on Si.

To evaluate the quality of the studied Gr samples AFM and micro-Raman measurements have been carried out. A typical result is reported in Figure 1 for the as-transferred Gr.

The AFM measurements (Figure 1a) show that Gr features islands of dimensions larger than 10 μm without any superimposed particles from the transfer process. The micro-Raman measurements show the typical bands associated with Gr centered at (2677 ± 1) cm^−1^ and at (1585 ± 1) cm^−1^ (Figure 1b). They are attributed to the 2D and G bands, respectively. No signal of the D band at about 1350 cm^−1^ is found. The amplitude ratio *I*_2D_/*I*_G_ of the 2D and G bands is 3.2, and a full width at half maximum (FWHM) of the 2D band of (35 ± 1) cm^−1^ was determined. On the basis of AFM and the micro-Raman measurements it can be concluded that the studied samples are monolayer Gr with a low number of defects [29–30].

**Figure 2:**
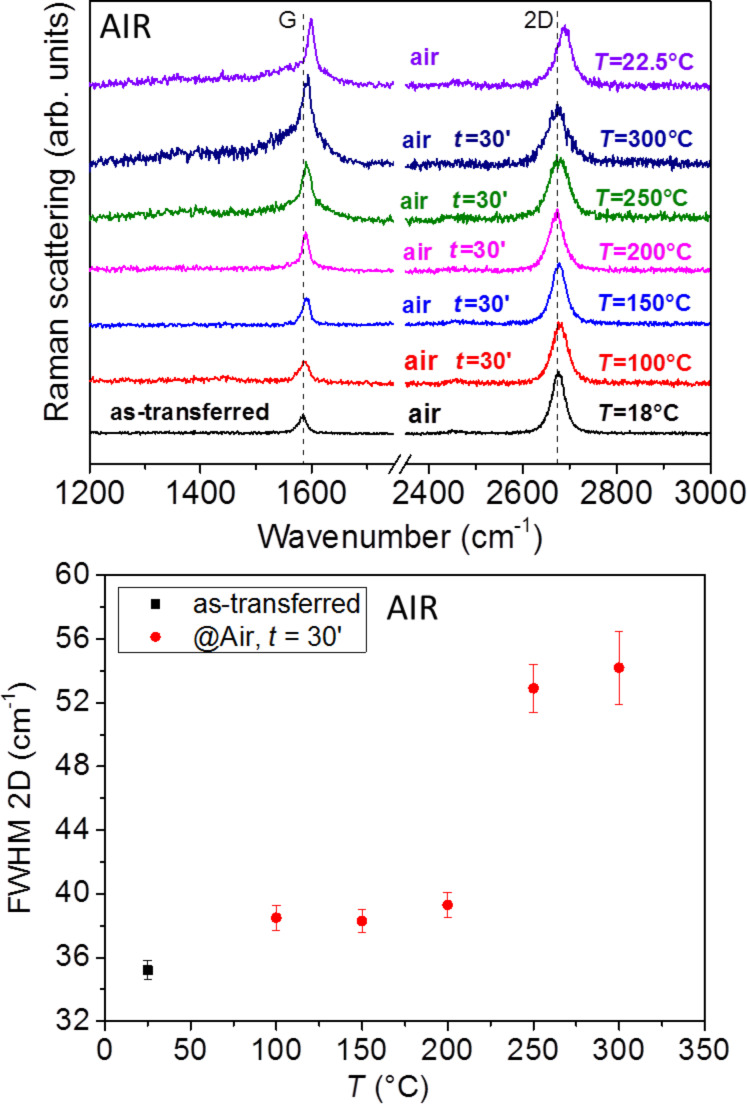
(Top) Comparison of the in situ Raman spectra of a Gr sample as transferred and subsequently treated in air, the temperature varies from 100 to 300 °C. The reported time is the elapsed time from the treatment beginning at the given temperature, the top spectrum is acquired after decreasing the temperature. Spectra have been arbitrarily vertically shifted for clearness. (Bottom) Measured FWHM of the 2D band as a function of the temperature.

To evaluate the effects of temperature and to follow the changes in the Raman spectrum during thermal treatments, we made two experiments with in situ measurements. The first experiment, reported in Figure 2, was carried out in air varying the temperature from 100 to 300 °C in 50 °C steps and maintaining each single temperature for 30 min during which Raman measurements were made every 5 min.

In Figure 2 we can see a clear increase in the FWHM of the 2D band with increasing temperature, which is attributable to thermal effects [31]. In detail, the FWHM features a constant increased value between 100 and 200 °C, and a further increase at 250 °C. The value returns to that of the as-transferred sample after cooling to sample to room temperature. There is no evident shift of the 2D band peak position, and only a small blue shift of the G band. Furthermore, we found the appearance of a shoulder at low wavenumbers near the G band above 200 °C. In addition, beginning at 150 °C we observed a change in the amplitude ratio of the 2D and G bands, *I*_2D_/*I*_G_, with a tendency to decrease. After cooling the sample to room temperature (top spectrum in Figure 2) there is a permanent blue shift of the G (13 cm^−1^) band and of the 2D (13 cm^−1^) band, and a decrease of their ratio from 3.4 down to 1.4 (the latter value has been evaluated subtracting a linear tangent to the G band bottom to take into account the shoulder and background induced by thermal treatments above 200 °C). The observed blue shift can be attributed to the doping by molecular oxygen present in the air atmosphere [19,21].

The second experiment with in situ measurements was made by thermal treatment in 2 bar of oxygen atmosphere at 300 °C for up to one hour and, successively, at 350 °C for an additional hour. This choice was determined by the above reported temperature value inducing major observable spectral changes and by the previously reported study about the doping effects as a function of temperature in similar samples, showing a larger effectiveness of doping at these temperatures [19].

**Figure 3:**
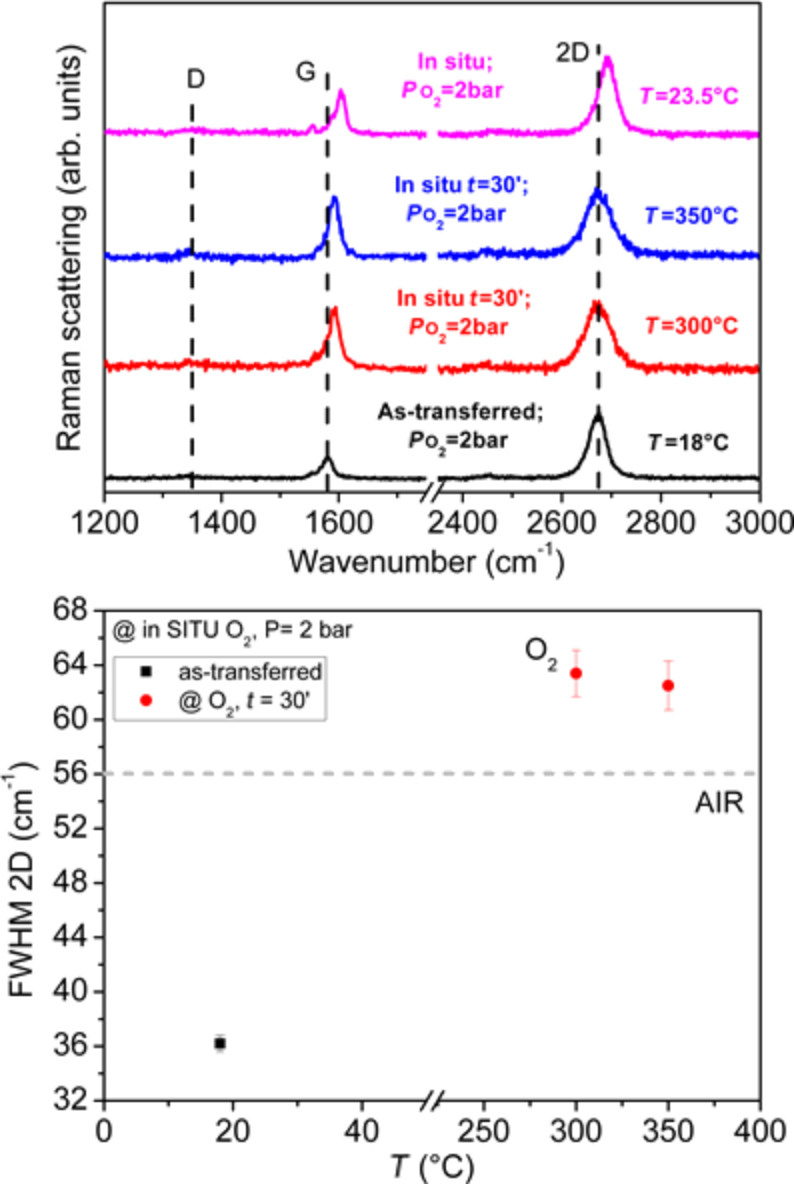
(Top) Comparison of the in situ Raman spectra of a Gr sample as transferred and subsequently treated in O_2_, at a pressure of 2 bar and at temperatures of 300 and 350 °C. The reported time is the elapsed time from the treatment beginning at the given temperature. Spectra have been arbitrarily vertically shifted for clearness. (Bottom) Trend of the FWHM of the 2D band as a function of the temperature. The dashed line indicates the maximum value of the FWHM of the 2D band obtained by the treatment in air (see Figure 2).

As shown in Figure 3, also in this case we see the thermal effect with a larger increase in the FWHM of the 2D band compared to that observed in the air, without a shift. Again a decrease of the *I*_2D_/*I*_G_ bands amplitude ratio down to 1.1 is observed. Also in this case, after cooling the sample to room temperature (top spectrum in Figure 3) a blue shift of the G and 2D bands (of 20.5 and 18 cm^−1^, respectively) is detected. The FWHM of the latter band returns to be like that recorded for the as-transferred sample. Compared to the sample treated in air, we note a greater shift of the G and 2D bands, indicating an additional doping effect induced by the exposure to pure oxygen [18–19].

These findings are compatible with those reported in our previous work [20], were a blue shift of both the G and 2D bands of 19 and 14 cm^−1^, respectively, was found. The differences to the treatment carried out in air could be partially due to the presence of other molecules in the atmosphere as can be deduced by the experiments carried out previously in similar systems [20]. Furthermore, on returning to room temperature we observed an increase of the *I*_2D_/*I*_G_ ratio probably caused by the high temperature that could induce the deterioration of graphene. In fact, in a previous work [19] it was shown that at 350 °C a removal of the edges of Gr flakes occurs, thereby suggesting that a strong structural change can be induced by this temperature.

**Figure 4:**
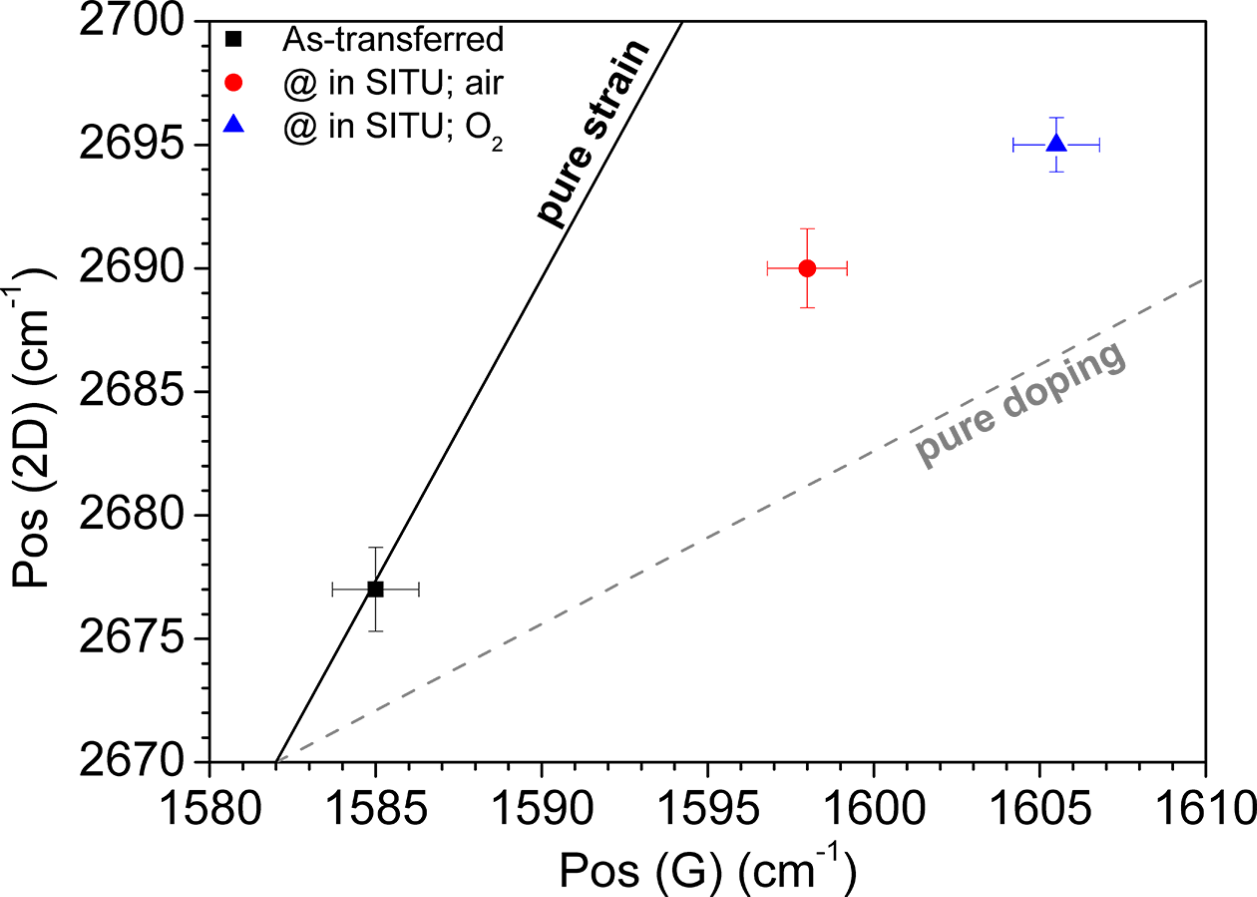
Correlation map of the 2D and G peak positions measured at room temperature in the Gr/SiO_2_/Si samples as transferred and thermally treated selectively in air and in O_2_. The full line marks the stress limit and the dashed line the doping limit as reported in [30].

Similarly to what is found in the literature [30], we report in Figure 4 a map of the 2D band position as a function of the G band position to evidence doping and stress effects. As shown in the figure, the sample that has undergone a treatment in oxygen is closer to the line of doping than to the strain line, obtained by a Raman spectrometer with 2.33 eV laser excitation. This supports the conclusion that p-type doping was obtained by treatment in O_2_. Furthermore, a comparison with previous results [21] reporting the effects of thermal treatments in a similar temperature range evidence that the effectiveness of doping found in the present work can be mainly attributed to the presence of oxygen molecules in the treatment atmosphere. Indeed, using other gases (N_2_, CO_2_, vacuum) gives points in a map analogous to the one in Figure 4 more confined to the “pure strain” line.

It has been recently reported that a temperature increase induces a red shift both of G and 2D bands as well as line broadening and the appearance of a shoulder in the G band due to thermal strain [31]. These features are compatible to the results reported here. In particular, the absence of a blue shift during in situ measurements and exposure to oxygen can be interpreted as a competition between a temperature-induced red shift and a doping-induced blue shift. This effect needs to be further investigated to be fully clarified and to disentangle mechanical and doping effects, for example by changing the gas composition during thermal treatments.

**Figure 5:**
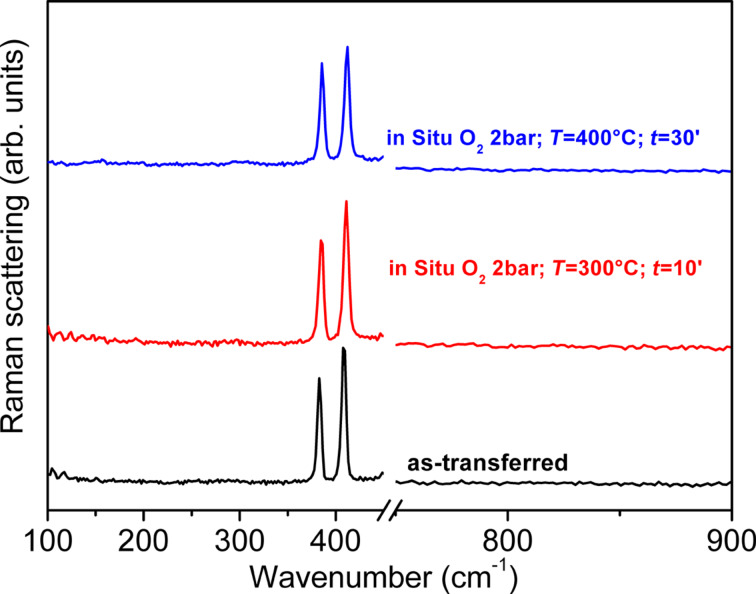
In situ Raman spectra of MoS_2_ before thermal treatments (bottom), after thermal treatment in O_2_ at 300 °C (middle) and 400 °C (top). Spectra are vertically shifted for the sake of comparison.

To investigate the effects of a similar oxygen treatment on MoS_2_, a sample has been treated in O_2_ atmosphere at 2 bar in the temperature range of 300–430 °C in a Linkam cell. In this temperature range doping should be effective for few layers as recently reported in literature [25]. An increase of temperature in steps of 10 °C was applied from 300 up to 390 °C and, at each step, the sample was maintained at the selected temperature for 10 min and Raman measurements were collected every minute. Afterward the sample temperature was increased up to 400 °C for 30 min, collecting Raman spectra. As reported in Figure 5, the Raman spectrum of the native sample features the typical bands of bulk MoS_2_ associated to the E^1^_2g_ and A_1g_ modes at 383 and 409 cm^−1^, respectively. This shows that the applied exfoliation process produced multilayer flakes. For all the investigated temperatures, the treatments give no detectable changes in the Raman band features. In particular we did not observe the appearance of the bands at 158, 285 and 820 cm^−1^ associated to MoO_3_, which is expected as side product of the oxygen thermally induced doping of MoS_2_ [25]. Nevertheless, during these treatments, we observed by microscope a gradual size reduction of the flakes. A further increase of temperature up to 430 °C had the same effects as lower temperatures.

**Figure 6:**
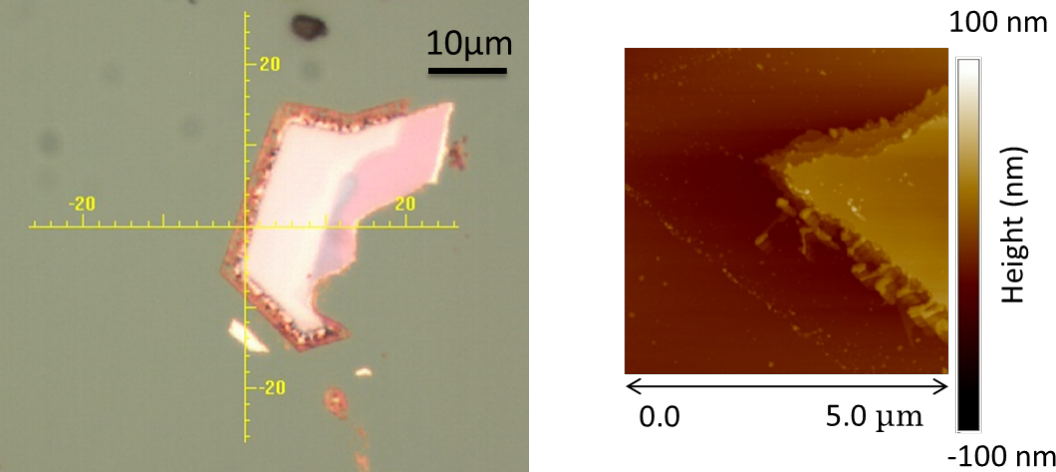
MoS_2_ flake after the thermal treatment up to 430 °C in O_2_; optical microscopy (left) and AFM (right) images.

As shown in Figure 6, we noticed a decrease of optical contrast in optical microscope measurements between the center of the MoS_2_ flakes and their edges, the latter appearing darker. To go deeper into this aspect we carried out AFM measurements and, as shown in Figure 6, we found that the edges of the flakes are thinner, suggesting the presence of a smaller number of layers.

**Figure 7:**
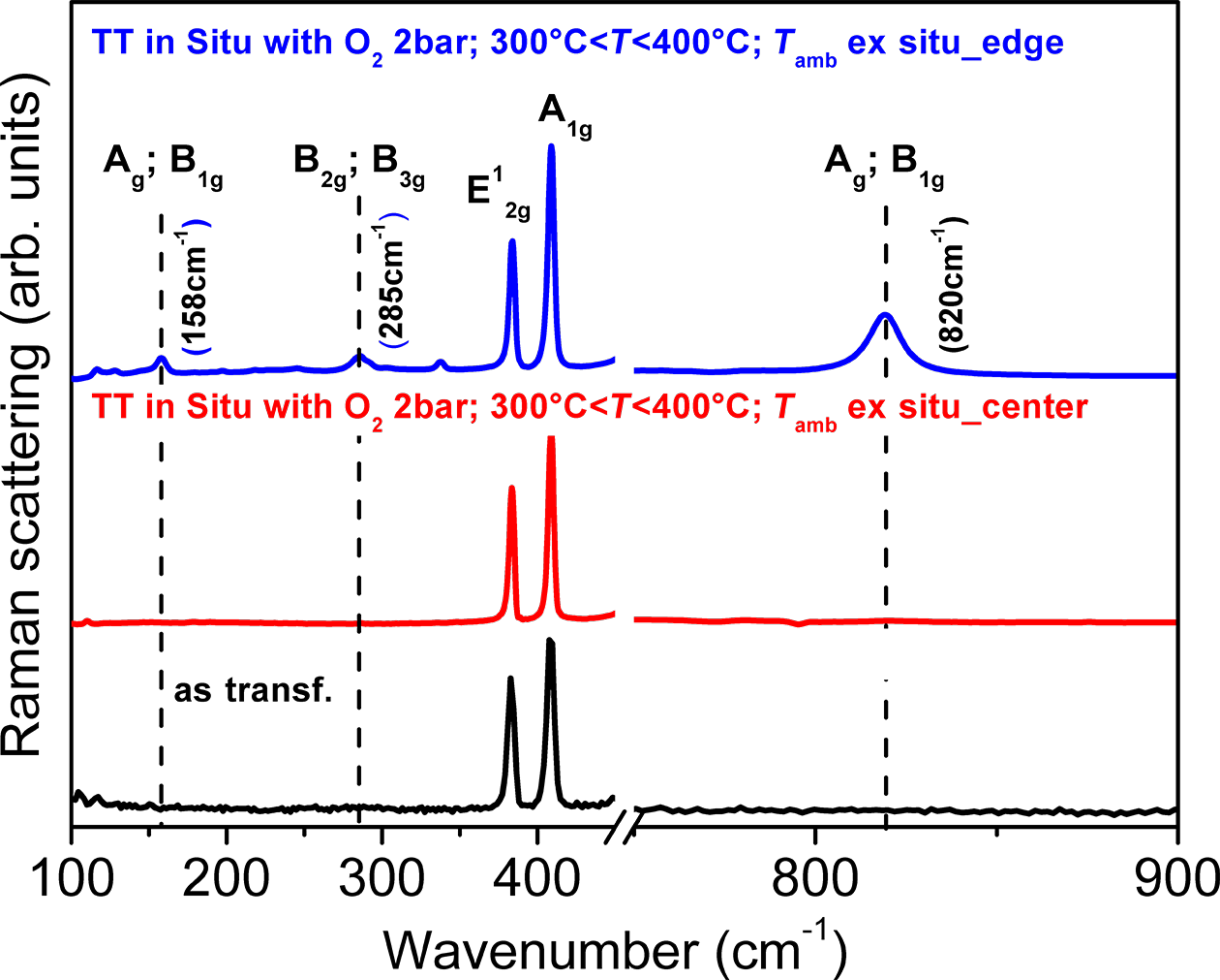
Ex situ Raman spectra of MoS_2_ before thermal treatment (bottom line), after thermal treatment in O_2_ at *T* < 430°C in the center of a flake (middle) and on the edge of a flake (top). Spectra are vertically shifted for the sake of comparison.

By carrying out Raman measurements ex situ (Figure 7), we found that the edges of the flakes have a different spectrum. In particular, the bands attributed to MoO_3_ at the spectral positions 158, 285 and 820 cm^−1^ are now clearly seen. This finding suggests that the observed erosion of the flakes is driven by the formation of oxidized regions at the borders of the MoS_2_ flakes that gradually convert them to MoO_3_, before completely destroying them. Similar effects have been suggested for other transition metal dichalcogenides and show that the thermal processes could be driven by analogous reactions [32]. In particular, these effects are of relevance for the application of 2D materials in ambient environments where the temperature could be increased during their use. Finally, the possibility that doping of central parts of the flakes occurred during the treatments cannot be excluded. But in order to obtain more information further in situ measurements with higher spectral resolution, which are able to detect shift or broadening of MoS_2_ Raman bands, are necessary.

## Conclusion

The experiments here reported on Gr samples transferred on SiO_2_ substrates have shown the possibility to monitor by in situ Raman measurements the effects of temperature and doping during thermal treatment in controlled atmosphere (air and oxygen). This study evidenced the competition in the Raman spectral changes between the thermal and the doping effects. In particular, the temperature increase induces a clear line broadening of the 2D band and masks the doping-induced shift of the G and 2D bands. At variance, doping effects can be monitored online through the change in the amplitude ratio of the G and 2D bands. Similar in situ measurements carried out to monitor O_2_-doping of MoS_2_ on SiO_2_ substrates have shown that the flakes produced by exfoliation are progressively destroyed at *T* > 300 °C through the generation of MoO_3_ on their edges, while mainly unchanged MoS_2_ remains in the central part.

## Experimental

The used samples are monolayer Gr and mechanically exfoliated MoS_2_ deposited on SiO_2_. The Gr samples were produced by chemical vapor deposition process on a Cu foil [11,19]. After Gr growth the foils were covered by PMMA and Cu was removed in a FeCl_3_ bath. Successively, a transfer process on a 300 nm thick SiO_2_ layer on Si following a well-established procedure has been carried out [11,19]. Micro-Raman measurements were carried out with a Horiba LabRam HR-Evolution spectrometer equipped with a 532 nm excitation laser. Raman measurements were carried out in situ, i.e., during thermal treatment, with the sample inserted inside a Linkam THMS600PS cell with temperature and pressure control, at a nominal laser output power of 25 mW and spectrometer spectral resolution of 7 cm^−1^ (600 lines per mm) to speed up acquisition time, after verifying that these parameters do not affect the Gr Raman signal. During the measurements the temperature was fixed up to 350 °C and the pressure up to 2 bar, when oxygen was used. The cell was purged twice at higher pressure to remove ambient atmosphere before sealing it and increasing the temperature. A heating ramp of 100 °C/min was selected, whereas cooling to room temperature was obtained within 25 min. Heating and cooling were executed in controlled atmosphere.

MoS_2_ samples were obtained by mechanical exfoliation of bulk natural molybdenite crystals, obtained from SPI (http://www.2spi.com). The exfoliation was based on thermal release tape and thermocompression printing on a SiO_2_ (300 nm) layer on Si substrate of the same kind used for Gr [11]. Flakes of lateral size larger than 10 μm, and thickness ranging from 10 to 100 nm were typically obtained by this procedure. In situ Raman measurements and thermal treatment up to 430 °C were carried out with the same equipment used for Gr. Furthermore, additional ex situ Raman measurements were carried out only on MoS_2_ with a Bruker Senterra micro-Raman spectrometer, with excitation at 532 nm, 5 mW output power and 9 cm^−1^ maximum spectral resolution (400 lines per mm).

Atomic force microscopy (AFM) measurements in air were executed by a Veeco DI3100 atomic force microscope with Nanoscope V controller working in tapping mode and employing a commercial silicon probe with spring constants of *k* = 20–80 N·m^−1^ and oscillation frequencies from 332 to 375 kHz.
